# Evaluation of Intercaval Bundle Connection by Multisite Pacing and Right-Sided Pulmonary Vein Isolation

**DOI:** 10.1016/j.jacadv.2025.102486

**Published:** 2025-12-29

**Authors:** Manabu Kashiwagi, Akio Kuroi, Daichi Miyake, Yusuke Teruya, Satoshi Hata, Natsuki Higashimoto, Chihiro Kansako, Yuto Otsuki, Yasutsugu Shiono, Takashi Yamano, Takashi Tanimoto, Hironori Kitabata, Atsushi Tanaka

**Affiliations:** a Department of Cardiovascular Medicine, Wakayama Medical University, Wakayama, Japan; b Clinical Engineering Center, Wakayama Medical University, Wakayama, Japan

**Keywords:** atrial fibrillation, catheter ablation, intercaval bundle connection, pulmonary vein isolation

## Abstract

**Background:**

An intercaval bundle connection (IBC) between the right pulmonary vein (PV) and the right atrium (RA) reduces PV isolation success, but the epidemiology requires elucidation.

**Objectives:**

The authors investigated the frequency and distribution of IBC diagnosed by multisite pacing and its relation to PV isolation outcomes.

**Methods:**

We analyzed 108 patients who underwent first PV isolation under radiofrequency ablation. An activation map was created by multisite pacing from the RA, the right inferior PV, and the earliest activation site of RA (EAS-RA) via right inferior PV pacing.

**Results:**

Compared with RA pacing, multisite pacing more frequently delineated IBC (100% [108/108] vs 20.4% [22/108]; *P* < 0.001). The EAS-RA was classified as “above” (n = 19), “below” (n = 79), and “far below” (n = 10) in relation to the right inferior PV. The earliest activation site of the left atrium (EAS-LA) obtained by EAS-RA pacing was classified as “PV inside” (n = 11), “PV side” (n = 15), and “LA side” (n = 81) in relation to the PV isolation line. The median distances from the EAS-LA to the PV ostium in the “above,” “below,” and “far below” groups were significantly different (3.4 [IQR: −2.1 to 7.2] mm vs 7.7 [IQR: 6.8-13.5] mm vs 15.2 [IQR: 13.4-20.7] mm; *P* < 0.001). Notably, “above” EAS-RA was related to “PV inside” EAS-LA. Additional ablation for the anterior carina was more common in “PV inside” than “PV side” and “LA side” (75.0% [9/12], 46.7% [7/15] and 6.2% [5/81]; *P* < 0.001).

**Conclusions:**

The IBC was visualized in all cases by multisite pacing. An attachment site on the LA side influenced the frequency of additional ablation for the right anterior PV carina.

Extended-circumferential pulmonary vein (PV) isolation is an established treatment for atrial fibrillation (AF).^1^ The efficacy and safety have been improved by the use of lesion index–guided ablation with contact force sensing catheters.^2^ However, circumferential PV isolation by radiofrequency catheter ablation is not always possible. Additional ablation for the PV inside may be required due to the presence of an intercaval bundle connection (IBC) between the right PV and the right atrium (RA).^3^, ^4^, ^5^, ^6^, ^7^, ^8^, ^9^

In addition to the Bachman’s bundle, the general route of electrical conduction from the RA to the left atrium (LA) during sinus rhythm, the presence of musculature bundle connection between the LA and posterior RA around the intercaval area has been confirmed in most cases.^10^^,^^11^ In cases with an epicardial connection from the RA to the right PV during sinus rhythm, additional ablation of the PV carina site is frequently required to complete PV isolation.^5^ In addition, this epicardial conduction to the right PV is reportedly associated with transient PV isolation and reconnection in the acute phase, and a higher risk of AF recurrence.^3^^,^^12^^,^^13^ Evaluations of IBC were previously conducted during sinus rhythm or pacing from the RA, and after PV isolation. Approximately 5 to 50% of cases were considered to have epicardial conduction of IBC.^4^, ^5^, ^6^, ^7^, ^8^ This is inconsistent with the previous autopsy result of >90%.^9^ Also, there is no uniform definition of the precise attachment site of the IBC; the actual in vivo frequency and distribution of IBC require confirmation. In the current study, we therefore investigated the frequency and distribution of IBC by conducting multisite pacing before PV isolation. We also examined the relationship between the distribution of IBC and the clinical outcomes of PV isolation.

## Material and methods

### Study population

We retrospectively analyzed the 108 consecutive patients who underwent first PV isolation under ablation index (AI)–guided radiofrequency ablation for AF at Wakayama Medical University between April 2023 and March 2025.^2^ During this period, all patients were basically evaluated for IBC by multisite pacing maneuver. The exclusion criteria were existence of incision line in the atrium due to previous cardiac surgery, the inability to stably conduct pacing atrium or PV, sustained AF rhythm even after left-sided PV isolation regardless of direct current cardioversion, and cases that operators otherwise judged to be inappropriate for inclusion. This study was carried out in accordance with the Declaration of Helsinki and it was approved by the Wakayama Medical University Research Ethics Committee (4,482). The requirement for written informed consent was waived because of the retrospective nature of the study.

### Ablation method

All patients were mildly sedated with dexmedetomidine hydrochloride and/or thiopental sodium.^14^ A 20-electrode atrial cardioversion catheter (BeeAT, Japan Lifeline) was inserted into the coronary sinus via the right internal jugular vein. After transseptal puncture under intracardiac ultrasonographic guidance, a multielectrode mapping catheter (OCTRAY, Biosense Webster) with long sheath (SL0, St. Jude Medical) and an irrigated ablation catheter (Thermocool Smarttouch SF, Biosense Webster) with deflectable sheath (VIZIGO, Biosense Webster) were placed in the LA via the right femoral vein. The specific strategies for catheter ablation for AF were decided by individual operators, but ipsilateral extended circumferential PV isolation under AI guide was basically performed. The target interlesion distance between 2 neighboring lesions was within 4 mm. The target AI values were 425-450, except for the posterior wall of the left PV (325-375) and the posterior wall of the right PV (400-425) with the power settings of 30 to 35 W. At esophageal lesions on the posterior wall, an esophageal temperature of <40 °C was maintained. When the right PV isolation was achieved, we did not apply additional anterior carina ablation based on the result of IBC mapping. We occasionally considered additional radiofrequency ablation for non-PV foci.

### Assessment of intercaval bundle connection

**Figure 1:**
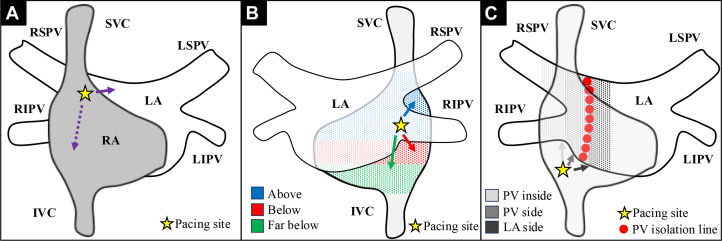
Schema of Pacing Diagnosis (A) Pacing from the RA. (B) Pacing from RIPV. Earliest activation site of the right atrium (EAS-RA) was classified as “above,” “below,” and “far below” based on location of the RIPV. (C) Pacing from EAS-RA obtained by RIPV pacing. Earliest activation site of the LA was classified as “PV inside,” “PV side,” and “LA side” based on location of PV and ablation line. IVC = inferior vena cava; LA = left atrium; LIPV = left inferior pulmonary vein; LSPV = left superior pulmonary vein; PV = pulmonary vein; RA = right atrium; RIPV = right inferior pulmonary vein; RSPV = right superior pulmonary vein; SVC = superior vena cava.

**Figure 2:**
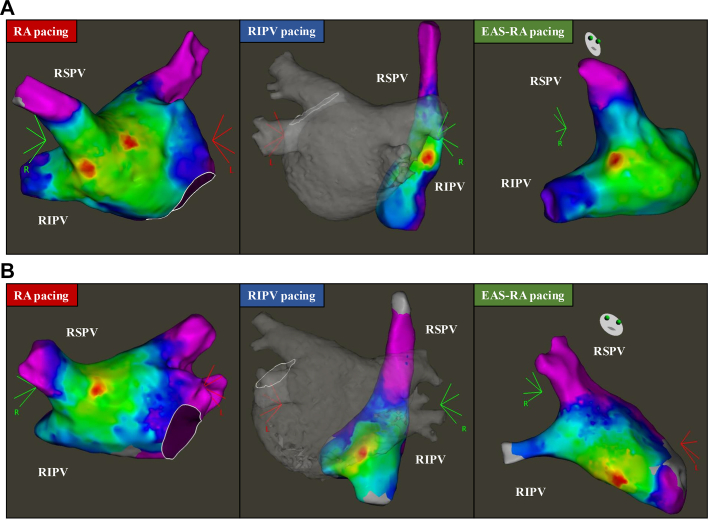
Multisite Pacing to Diagnose Intercaval Bundle Connection (A) In case 1, additional early activation site was observed around the right pulmonary vein (PV) during right atrium (RA) pacing. Earliest activation site of the right atrium (EAS-RA) was observed above the bottom of the right PV during right inferior pulmonary vein (RIPV) pacing. Earliest activation site of the left atrium (EAS-LA) was observed on the PV side compared to the PV isolation line during EAS-RA pacing. (B) In case 2, additional early activation site was not observed during RA pacing. EAS-RA was observed below the bottom of the right PV. EAS-LA was observed in the left atrium side compared to the PV isolation line. Abbreviation as in Figure 1.

If AF rhythm could be sustained during assessment of IBC, we attempted intracardiac direct current cardioversion. If sinus rhythm could not be sustained, regardless of intracardiac direct current cardioversion, we conducted left-sided PV isolation and then attempted intracardiac direct current cardioversion again. The activation map was made using CARTO ConfiDENSE module (Biosense Webster), and annotation points were evaluated by 2 independent investigators so to not include far-field potential, pacing spike, or noise. We performed the following pacing procedure to evaluate IBC, including pacing from RA, right inferior PV, and earliest activation site of RA (EAS-RA) obtained by right inferior PV pacing. The schema and representative cases are shown in Figures 1 and 2.

#### Pacing from high RA

First, pacing was attempted from the RA (the earliest activation site during sinus rhythm of a 20-electrode atrial cardioversion catheter placed in the coronary vein), and the LA activation map was created with a multielectrode mapping catheter (Figure 2A). If an additional early activation site was observed around the right PV that differed from the Bachman’s bundle, it was considered to be an IBC (Figure 2D).

#### Pacing from right inferior PV

**Figure 3:**
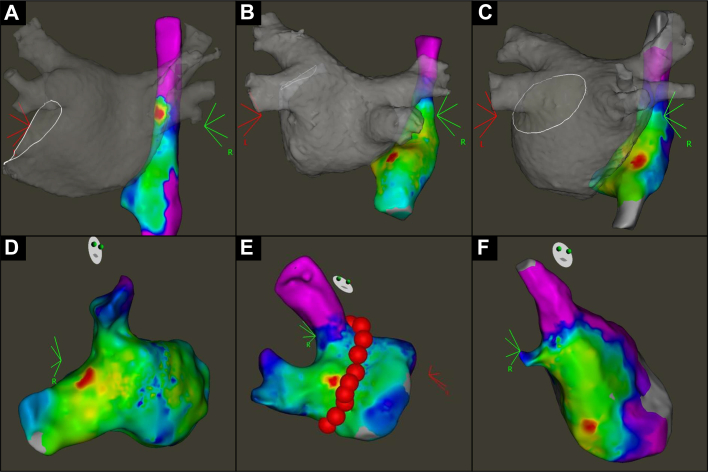
Classification of Attachment Site of Intercaval Bundle Connection (A) Above. (B) Below. (C) Far below. (D) Pulmonary vein inside. (E) Pulmonary vein side. (F) Left atrium side.

Next, pacing was performed from the bottom of the right inferior PV using an ablation catheter, and the RA activation map was created using a multielectrode mapping catheter to identify the EAS-RA. The EAS-RA during right inferior PV pacing was classified according to the location of the bottom of the right PV (“above” or “below”) (Figures 3A to 3C). If EAS-RA was further than the diameter of one right inferior PV, it was classified as “far below.”

#### Pacing from earliest activation site of right atrium

Lastly, we performed pacing by an ablation catheter from the EAS-RA obtained by right inferior PV pacing and created the activation map of LA around the right PV area by multielectrode mapping catheter. When multiple EAS-RAs were found by pacing from the right inferior PV, we selected the EAS-RA closer to the head (Supplemental Figure 1). For the earliest activation site of the LA (EAS-LA) obtained by EAS-RA pacing, we evaluated the distance from the ostium of PV. If the EAS-LA was within the PV, the distance was expressed as a negative value. For the PV isolation line, the distance from the ostium of the PV was evaluated at the cross point between the PV isolation line and the line from the PV ostium to the EAS-LA. The EAS-LA was classified as “PV inside,” “PV side,” and “LA side” according to the locational relationship between PV ostium, EAS-LA, and PV isolation line (Figures 3D to 3F).

The anatomical location of the bottom and ostium of the right PV were evaluated based on preoperative computed tomography (CT). For the CT merge, the location of CT information was finally manually adjusted based on the intraoperative electrical mapping and lesion tag information.

### Statistical analysis

Statistical analysis was performed using JMP Pro version 17.0 for Macintosh (SAS Institute) and R version 4.5.2 (R Foundation for Statistical Computing). Results are expressed as median (IQR), and qualitative data are as numbers and percentages. The nonparametric Mann-Whitney *U* test was used to test for differences between the groups. Fisher exact test was applied for categorical variables. The receiver-operating curve (ROC) was used to determine the best cutoff value of distance between the PV ostium and the PV isolation line for predicting additional ablation at anterior PV carina. The best cutoff value was determined according to maximum Youden’s index. Cox proportional hazards regression analysis was used to test the association of EAS-LA and recurrence of any atrial tachycardia. The proportional hazards assumptions of the Cox proportional hazard model were confirmed using the Schoenfeld residual test, and the Cox proportional hazards model presented here met the assumption of proportional hazards. The risk of recurrence of any atrial tachycardia was expressed as HR, 95% CI, and *P* value. *P* < 0.05 was considered to be statistically significant.

## Results

### Patient characteristics and ablation method

We intended to perform the multisite pacing maneuver in 138 patients. In 26 patients, AF rhythm was sustained even after left PV isolation and subsequent direct current cardioversion, and we were unable to conduct stable pacing in 4 patients. Patient characteristics are summarized in Table 1. The median age was 69 (IQR: 60–74) years, and 63.9% (69/108) were men. PV isolation was successful in all patients (Table 2). The median total procedural time was 94 (IQR: 80–118) minutes and 45 (IQR: 40–57) minutes for PV isolation. In addition to PV isolation, we also performed left atrial roofline ablation (n = 1), cavotricuspid isthmus linear ablation (n = 17), cryoablation for atrioventricular reentrant tachycardia (n = 1), and trigger ablation for non-PV foci (n = 2). No serious complications, such as embolism or pericardial effusion, occurred in any of the patients.

**Table 1:** Baseline Clinical Characteristics (N = 108)

Age, y	69 [60-74]
Men	69 (64)
BMI, kg/m^2^	24.3 [22.1-27.3]
Comorbidity	
Hypertension	74 (69)
Diabetes mellitus	21 (23)
Heart failure	20 (19)
Stroke	9 (8)
Estimated glomerular filtration rate, mL/min/1.73 m^2^	57.4 [46.7-66.8]
Echocardiographic data	
LV diastolic dimension, mm	46 [43-50]
LV systolic dimension, mm	31 [27-35]
IVS, mm	9 [9-10]
PW, mm	9 [8-10]
Ejection fraction, %	57 [52-59]
Left atrial diameter, mm	41 [37-46]
Mean E/e' ratio	9.2 [7.3-11.0]

Values are median [quartiles] or n (%).

BMI = body mass index; IVS = interventricular septum; LV = left ventricular; PW = posterior wall.

**Table 2:** Catheter Ablation Data

Procedural Time	
Total (skin to skin), min	94 [80-118]
Pulmonary vein isolation, min	45 [40-57]
Pulmonary vein isolation success	108 (100)
First-pass right-sided pulmonary vein isolation	71 (66)
Additional ablation for anterior right pulmonary vein	21 (19)
Additional ablation	
Left atrial roof line	1 (1)
Cavotricuspid isthmus line	17 (16)
Trigger ablation for atrial fibrillation	2 (2)
Slow pathway ablation	1 (1)
Serious complication	0 (0)

Values are median [quartiles] or n (%).

### Location of earliest activation site by multiple sites pacing maneuver

In all patients, early activation sites via the Bachman’s bundle were observed in the anterior wall of the LA with a median pacing interval of 48 (IQR: 39–58) ms. In 22 patients (20.4%), additional early activation sites of LA were also observed around the right PV area with a median pacing interval of 58 (IQR: 46–72) ms, which was later than the conduction via Bachman’s bundle in all cases.

The locations of EAS-RA obtained by the right inferior PV pacing were “above” the bottom of the right inferior PV in 19 patients, “below” in 79 patients, and “far below” in 10 patients. There was no difference in the pacing interval at EAS-RA between the “above,” “below,” and “far below” groups (49 [IQR: 42-57] ms vs 46 [IQR: 38-54] ms vs 47 [IQR: 42-55] ms, *P* = 0.791). The EAS-RA obtained by right inferior PV pacing was located around the intercaval bundle area in all cases. In this study, the highest EAS-RA was at the level of the carina of the right PV, and the EAS-RAs of all cases were anatomically far from the coronary sinus.

**Figure 4:**
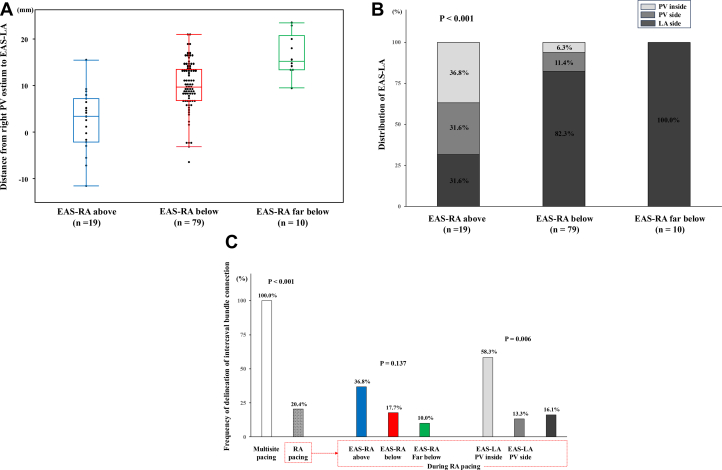
Frequency and Distribution of Earliest Activation Site (A) Relationship between distance from right pulmonary vein ostium to earliest activation site and earliest activation site of the right atrium. (B) Relationship between location of earliest activation site of right and left atrium. (C) Frequency of delineation of intercaval bundle connection during multisite pacing vs right atrial pacing. EAS-RA = earliest activation site of right atrium; EAS-LA = earliest activation site of left atrium; other abbreviations as in Figure 1.

In all cases, the location of EAS-LA obtained by EAS-RA pacing was different from the EAS-LA obtained by RA pacing, which was conducted via the Bachman’s bundle and observed in the anterior wall of the LA. The EAS-LA obtained by EAS-RA pacing was located in median 9.3 [IQR: 6.3–13.6] mm away from the right PV ostium, and the EAS-LA in “PV inside,” “PV side,” and “LA side” were observed in 12 (11.1%), 15 (13.9%), and 81 (75%) patients, respectively. The distance from the EAS-LA to PV ostium in the “above,” “below,” and “far below” groups were significantly different (3.4 [IQR: −2.1 to 7.2] mm vs 7.7 [IQR: 6.8-13.5] mm vs 15.2 [IQR: 13.4–20.7] mm; *P* < 0.001) (Figure 4A). In addition, the location of EAS-LA was statistically different between the 3 groups (*P* < 0.001) (Figure 4B). Although “above” group was equally related with “PV inside,” “PV side,” and “LA side” EAS-LA, the “far below” group was related with only “LA side” EAS-LA.

Compared with RA pacing, multiple site pacing more frequently delineated early activation site around right PV (100% [108/108] vs 20.4% [22/108]; *P* < 0.001) (Figure 4C). Although location of EAS-RA was not related (*P* = 0.137), additional early activation site during RA pacing was more frequently delineated in patients with EAS-LA in PV inside (*P* = 0.006).

### Earliest activation site and catheter ablation result

The distance from the right PV ostium to PV isolation line was 5.5 (IQR: 4.1–7.0) mm. There was no significant difference in the distance from the PV ostium to the PV isolation line among the EAS-RA “above,” “below,” and “far below” groups (5.9 [IQR: 4.2–7.5] mm vs 5.5 [IQR: 4.1–7.0] mm vs 5.2 [IQR: 4.1–6.5] mm; *P* = 0.845).

**Figure 5:**
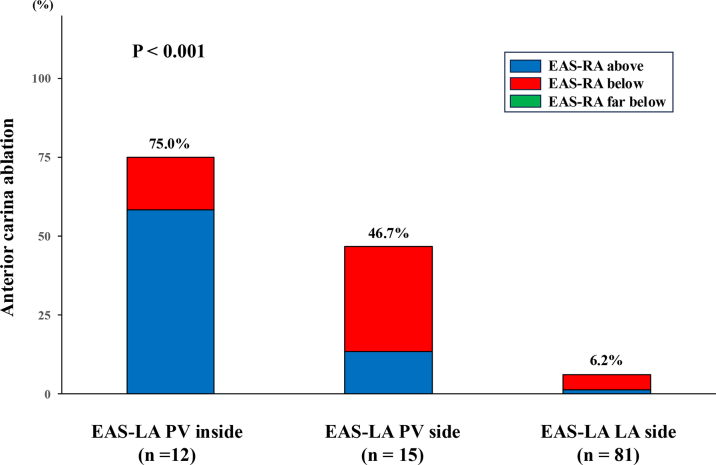
Additional Anterior Carina Ablation Relationship between frequency of anterior carina ablation and location of early activation site of left atrium. Abbreviations as in Figures 1 and 4.

Frequencies of additional ablation for the anterior carina of right PV were 75.0% (9/12), 46.7% (7/15), and 6.2% (5/81), respectively (*P* < 0.001) (Figure 5). There were no cases with “far below” EAS-RA requiring additional anterior carina ablation. For the ROC, the best cutoff value of the distance from the PV ostium to EAS-LA minus those to the PV isolation line to predict additional anterior carina ablation was −2.0 mm, with an area under the curve of 0.87 (95% CI: 0.77-0.96), sensitivity of 0.76 (95% CI: 0.53-0.92), specificity of 0.92 (95% CI: 0.84-0.97), positive predictive value of 0.70 (95% CI: 0.47-0.87), and negative predictive value of 0.94 (95% CI: 0.87-0.98) (Supplemental Figure 2).

During follow-up (median period of 245 [IQR: 113-370] days), recurrences of atrial arrhythmia were observed in 33.3% (4/12) of cases with “PV inside,” 33.3% (5/15) of cases with “PV side,” and 25.9% (21/81) of cases with “LA side.” Cox proportional hazards regression analysis revealed that the location of EAS-LA was not related to recurrences of any atrial tachycardia (PV side: HR: 0.98; 95% CI: 0.33-2.88; PV inside: HR: 1.56; 95% CI: 0.58-4.20; LA side: reference; *P* = 0.695). Repeat catheter ablation was conducted in 33.3% (n = 4) with “PV inside,” 20% (n = 3) with “PV side,” and 19.8% (n = 16) with “LA side.” In the repeat session, the reconnection of right PV was observed in 16.7% (n = 2), 13.3% (n = 2), and 12.4% (n = 10), respectively.

## Discussion

In this study, an IBC from the posterior RA to the right PV area was observed in all cases by multisite pacing maneuver from right inferior PV and by EAS-RA obtained by right inferior PV pacing. When the EAS-RA was near the right inferior PV, the EAS-LA by pacing from EAS-RA was also near the right PV ostium. In addition, the relationship of anatomical location between the EAS-LA and the PV isolation line influenced the frequency of additional ablation of the anterior carina of the right PV.

### Epidemiology of intercaval bundle connections

**Figure 6:**
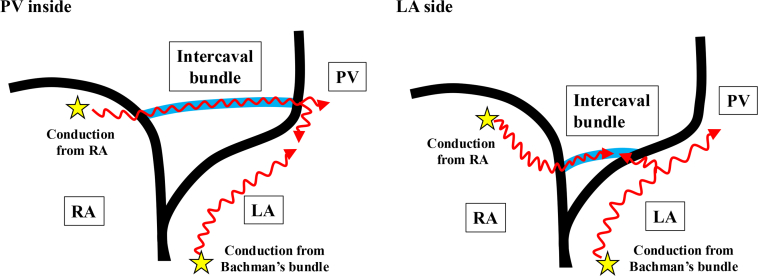
The Schema of Conduction From Right Atrium to Right Pulmonary Vein Abbreviations as in Figure 1.

The main electrical conduction route from the sinus node to the LA is generally the Bachman’s bundle, which is a wide musculature bundle extending from the junction of the superior vena cava and the RA, through the anterior wall of the LA, and finally to the area around the left atrial appendage.^10^^,^^11^ In autopsy-based studies, in addition to the Bachman’s bundle, musculature bundles from the coronary sinus to the inferior LA and IBC between the right PV and the posterior RA have been confirmed.^10^ IBCs were reportedly observed in approximately 90% of cases, but only a relatively small number of autopsies were performed. In addition, it is unclear whether this musculature bundle possesses electrical conduction ability. One study evaluated propagation of LA excitation by a three-dimensional mapping system, and they clarified that the additional early activation area around the right PV during sinus rhythm was observed in approximately 20% of cases. Additional ablation for the carina of the right PV was frequently required to achieve PV isolation in such a case.^5^ In another report, evaluation during RA pacing instead of sinus rhythm revealed an additional early activation site around the right PV in approximately 30% of cases, which authors concluded to be an epicardial connection. These results were consistent with those in the present study, where we found that 20.4% had an epicardial connection. The evaluation was performed during sinus rhythm or RA pacing in previous reports, so the main electrical conduction route from the RA to the LA was suggested to be the Bachman’s bundle, and the actual frequency of IBC was not evaluated. In the current study, an early activation site at intercaval area of RA was observed by right inferior PV pacing, which was different from the Bachman’s bundle and coronary sinus musculature pathway. By pacing from the early activation site at intercaval area of RA, contralateral early activation site was observed around the right PV. These conductions, which were thought to be an IBC, were observed in all of the 100+ cases. In cases with an IBC, in which attachment of the inside of right PV was confirmed by EAS-RA pacing, the IBC was frequently delineated, even during RA pacing. This may be because the IBC might be a shorter route than the Bachman’s bundle (Figure 6).

### Relationship between intercaval bundle connection and pulmonary vein isolation

When the delineated site of the IBC on the RA side was above, the IBC on the LA site was closer to the right PV ostium or PV inside. In addition, when the attachment point of the IBC was found on the PV side from the right PV isolation line, additional ablation for anterior carina inside the PV isolation line was conducted in more than half of the cases. Previous reports have shown that the gaps of extended circumferential PV isolation at carina region are presumed to be due to the thickening of the atrial wall.^15^ Recently, the lesion index has made it possible to accurately evaluate the extent of lesion formation.^2^ Our results suggest that the epicardial bundle conduction between the RA and the right PV is perhaps the main reason for unsuccessful right-sided PV isolation with AI guide, rather than the incomplete transmural lesion formation. In another study, a PV isolation line >8 mm away from the PV ostium was a predictor for failure in circumferential PV isolation.^14^^,^^16^ In this current study, the median distance from the PV ostium to the LA site of the IBC was 9.3 mm, which is thought to be consistent with the previous result.

In our study, PV isolation occasionally succeeded, even in the case that the LA site of IBC was located at the PV side in relation to the PV isolation line. In such a case, the IBC in the epicardial site could be ablated.^9^ However, these isolations could be temporary and demonstrate reconnection.^12^^,^^13^ The AF recurrence rate is reportedly high in cases with epicardial conduction.^3^ Careful ablation around the PV carina site might therefore be required, regardless of the risk for PV stenosis and phrenic nerve injury.

### Clinical implications

Evaluation of the attachment site of the IBC is important for effective PV isolation. However, we suggest our method would be complicated and time-consuming to evaluate all cases. In a previous report, IBC could be detected by assessment of intra-atrial sequencing and interval time from the right PV to the high RA during right PV pacing.^6^ In another, evaluating the difference between sinus rhythm and coronary sinus pacing was also said to be a simple and effective method to determine the existence of IBC.^8^ When extended circumferential PV isolation by radiofrequency ablation fails, these pacing maneuvers would be useful.

To date, pulsed field ablation has been developed as an alternative to radiofrequency ablation and balloon ablation.^17^ Some cases in the current study showed IBC to the ostial area of the right PV, so we suggest that acknowledgment of IBC might be helpful for a point-by-point system type of pulsed field ablation.^18^

In addition, IBC can be a circuit and therapeutic target for bi-atrial tachycardia.^19^^,^^20^ The Bachman’s bundle and coronary sinus musculature often have multiple connections to the LA, making complete transection difficult. Understanding and clarifying the presence of IBC is thus essential for treating complex bi-atrial tachycardia.

### Study limitations

The number of cases in this study was relatively small, so in a larger cohort, there might be cases in which an IBC does not exist. Also, patients with unstable pacing were excluded from this study, so the presence or absence of an IBC was not evaluated in these cases. Furthermore, only the earliest activation site was evaluated this time, so it is unclear whether there were multiple attachment sites or widespread attachment sites. In some cases, we do not know whether additional ablation at the anterior carina of right PV was really necessary. In particular, because reevaluation was not performed after PV isolation, the change in the earliest activation site after PV isolation could not be evaluated in cases with an IBC within the PV. In some cases, conduction via the Bachman's bundle might be misdiagnosed as IBC. Although pacing was attempted at a voltage as low as possible from the EAS-RA before PV isolation, we cannot deny the possibility of direct stimulation of the LA side. In addition, there might be incorrect or inaccurate annotation of the mapping point. Overdiagnosis and/or misclassification might therefore exist. Also, pacing was from the right inferior PV, so it is possible that epicardial conduction attached to the right superior PV area was underdiagnosed.

## Conclusions

The IBC from the RA to the right PV area could be visualized in all of our cases by multisite pacing maneuver, and its attachment to the right PV inside was observed in approximately 10% of them. The relation between the attachment site of the IBC on the LA side and the location of the right-sided PV isolation line influenced the additional ablation for anterior carina of the right PV (Central Illustration).

Perspectives **COMPETENCY IN MEDICAL KNOWLEDGE:** IBCs were observed in all cases, and attachment to the PVs occurred in approximately 10% of cases. The relationship of anatomical location between the attachment site on the left atrial side and the PV isolation line influenced the frequency of additional ablation of the anterior carina of the PV. Failure of right PV isolation under lesion-index guide is most likely due to IBC rather than insufficient lesion formation.

**TRANSLATIONAL OUTLOOK:** Multicenter and prospective studies with large cohort are required to determine therapeutic strategy for patients with IBC to PV.

**Central Illustration:**
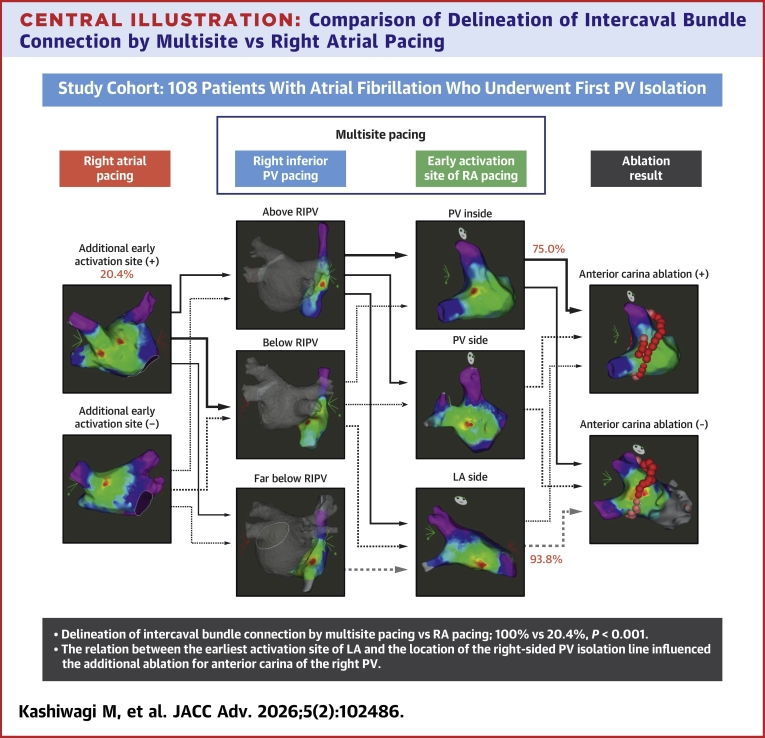
Comparison of Delineation of Intercaval Bundle Connection by Multisite vs Right Atrial Pacing In all cases, multisite pacing, consist of right inferior pulmonary vein (PV) pacing and earliest activation site of right atrium pacing, delineated the attachment site of intercaval connection. The location of earliest activation site of the left atrium was related additional ablation for anterior carina right PV. Abbreviations as in Figure 1.

## Funding support and author disclosures

This work was supported by 10.13039/501100001691JSPS 10.13039/501100001691KAKENHI Grant Number 24K11246. The authors have reported that they have no relationships relevant to the contents of this paper to disclose.
