# Supplementary figures and images for: Evaluation of Intercaval Bundle Connection by Multisite Pacing and Right-Sided Pulmonary Vein Isolation
Source: JACC Adv. 2025 Dec 29;5(2):102486. doi: 10.1016/j.jacadv.2025.102486 (PMC12804045; doi:10.1016/j.jacadv.2025.102486)

Supplementary Figure 1

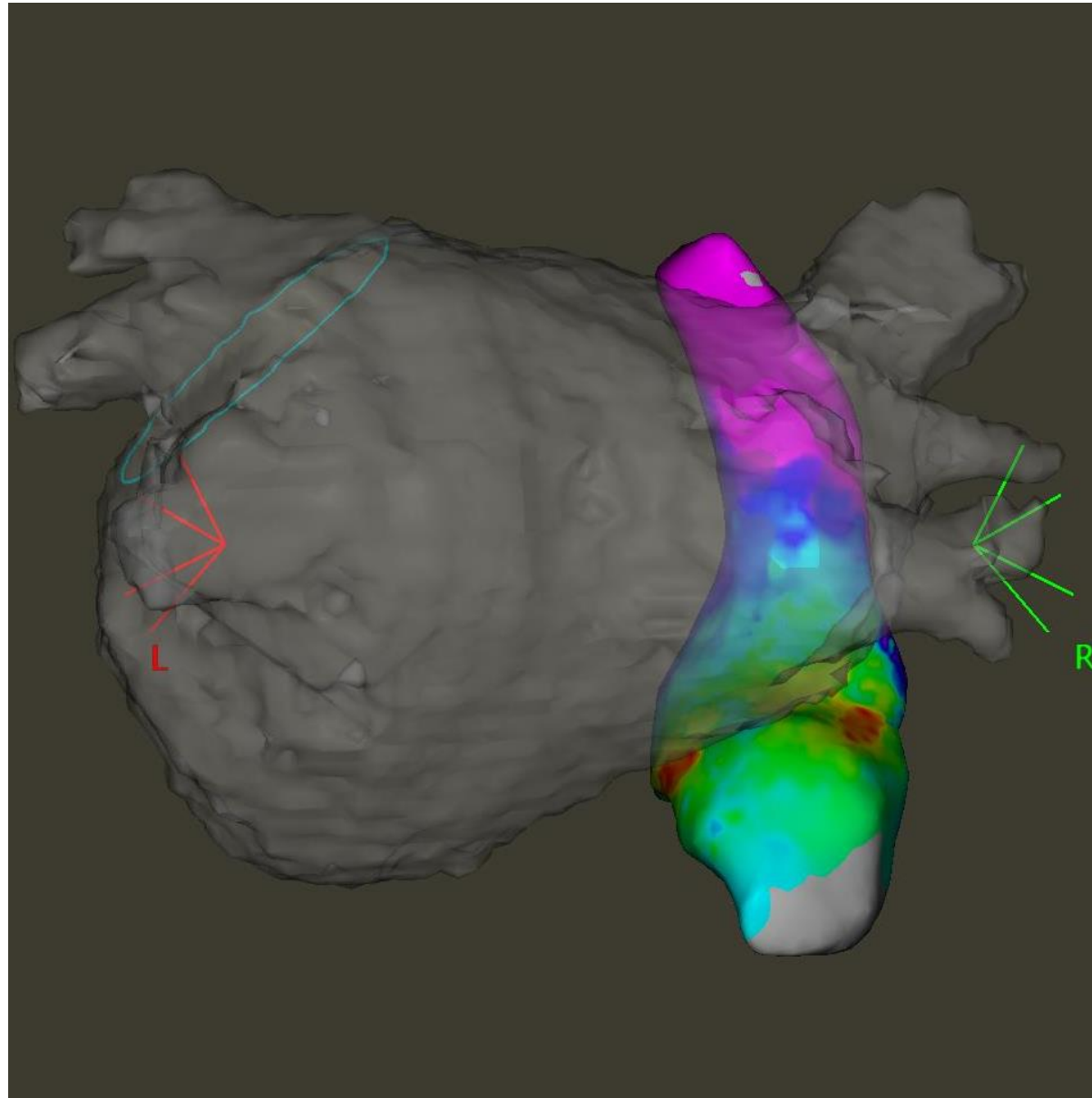

Supplementary figure 2

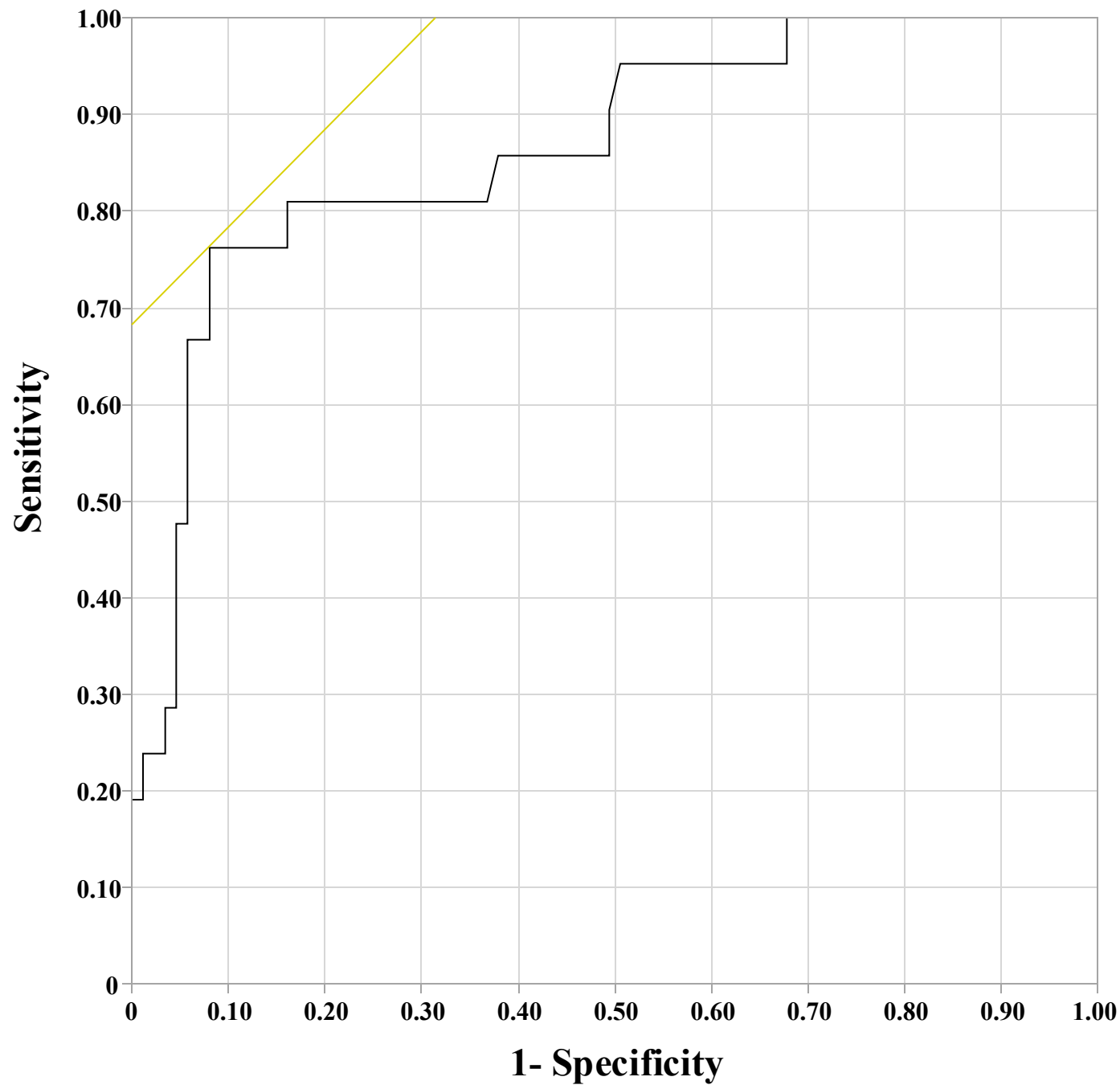

Supplement: Supplemental_Figures [file mmc1.pdf]
